# In Vitro Growth of Mammalian Follicles and Oocytes

**DOI:** 10.3390/ani14091355

**Published:** 2024-04-30

**Authors:** Kenichiro Sakaguchi

**Affiliations:** 1 Laboratory of Veterinary Theriogenology, Joint Department of Veterinary Medicine, Faculty of Applied Biological Sciences, Gifu University, Yanagito 1-1, Gifu 501-1193, Japan; sakaguchi.kenichiro.x3@f.gifu-u.ac.jp; 2 Division of Animal Medical Science, Center for One Medicine Innovative Translational Research, Institute for Advanced Study, Gifu University, Yanagito 1-1, Gifu 501-1193, Japan

Mammalian ovaries contain a large number of immature follicles, most of which are destined to degenerate before ovulation [1]. Developing a culture system that enables small oocytes in these follicles to grow to the stage where they can be fertilized in vitro would be a significant achievement that can be of great help in assistant reproductive technology, the production of farm animals, and experimental models for folliculogenesis and oogenesis [2,3].

In 2016, the generation of offspring from murine primordial germ cells (PGCs) using an entire in vitro growth (IVG) culture system was reported [4]. The birth of pups from iPS-cell-derived oocytes differentiated in vitro became reality in a murine model in the same year [5]. Now, it is expected that the application of such technology to livestock and endangered animals may no longer be just a dream for the future.

On the other hand, no offspring has been reported from secondary follicles or earlier stages using entire in vitro culture systems [6]. Although PGC-like cells were established in some species [7,8,9,10,11,12,13,14,15,16,17], the question of “how to foster eggs in vitro” remains the greatest challenge.

There are several aspects of the efforts employed to improve culture systems, and these include the preparation of ovarian tissues [18], isolation of follicles and oocytes [19,20], design of culture plates [21,22,23], creation of a matrix for media [24,25,26], and inclusion of supplements into media [27]. Additionally, the study of in vivo follicular development [28] and related signaling pathways [29,30] can be the best means of achieving reconstitution of the follicular environment in living animals.

The aim of this Special Issue was to present recent research in the development of the IVG of immature follicles and oocytes in mammalian species, as well as its application in stem cell technologies and utilization in experimental models.

Modak et al. [31] studied the effect of L-carnitine, a naturally produced important chemical for lipid metabolism, on the growth of oocytes in early antral follicles from water buffaloes, which have relatively smaller ovarian reserves [32]. In their study, oocytes cultured with L-carnitine (2.5 mM) showed higher growth and maturational competence.

Borș et al. [33] applied platelet-rich plasma (PRP) treatment [34] for bovine ovum pick-up (OPU)-in vitro fertilization (IVF) by injecting lyophilized horse-platelet-derived growth factor (L-GFequina) into ovaries. They suggested that the protocols improved follicular development and embryo productivity, which may imply possible application for immature oocytes in smaller follicles.

Nascimento et al. [35] tested the effects of N-acetylcysteine (NAC), an antioxidant [36], on the IVG of secondary follicles in cattle. Secondary follicles cultured with 1 mM of NAC showed better growth and differentiation to the antral stage, whereas oocytes inside of the follicles still showed signs of degeneration at the ultrastructure level. They suggested possible efforts could be some combination of antioxidants protecting not only surrounding granulosa cells but also oocytes.

de Assis et al. [37] also focused on an antioxidant, *Cimicifuga racemosa* (L.) Nutt extract (CIMI) [38], to investigate its protective effects on mouse ovaries cultured with Doxorubicin, a drug for cancer treatment with gonadotoxic side effects. They showed that CIMI protected follicles from Doxorubicin-induced apoptosis by reducing oxidative stress, which might be applied for fertility preservation in patients with cancer.

Sakaguchi et al. [39] investigated the metabolism of amino acids (which assume diverse roles such as protein synthesis, sources of energy and osmotic pressure, and pH adjustment for cell cultures [40]) during IVG to find non-invasive bio markers to predict the health status of follicles. They found possible molecular species that could be the marker for both cortical strip culture for primordial follicular activation (methionine, lysine, arginine, and α-aminoadipic acid) and IVG for secondary follicles (methionine, tyrosine, histidine, and hydroxyproline).

Kim et al. [41] examined various growth factors (epidermal growth factor (EGF), insulin-like growth factor-1 (IGF-1), insulin, and growth hormones) on IVG culture during the pre-maturation period of oocytes from small antral follicles (<3 mm), which have lower developmental competence then those derived from larger follicles [42]. Their results suggested that insulin supplementation (1 μg/mL) improved developmental competence in relation to the reduction in oxidative stress, and the productivity of embryos became comparable to their in vivo grown counterparts after 2 days of IVG.

In conclusion, this Special Issue covers culture systems from a wide range of follicular developmental stages, including primordial follicles [39], secondary follicles [35,39], early antral follicles [31], small antral follicles [41], in vivo grown antral follicles [35], and whole ovarian cultures [37]. Moreover, it consists of studies of four different species, including water buffaloes [31], cattle [33,35,39], mice [37], and pigs [39]. This variety suggests the need to study oocytes from early developmental stages in varied fields, as well as the importance of the development and refinement of culture systems for each follicle’s developmental stage.

In closing, I would like to acknowledge all the authors for their contributions to this Special Issue, and I hope this Special Issue can provide some insights in order to improve current culture systems and promote the wide application of the IVG of eggs across species.
